# Targeted genomic landscape of metastases compared to primary tumours in clear cell metastatic renal cell carcinoma

**DOI:** 10.1038/s41416-018-0064-3

**Published:** 2018-04-20

**Authors:** Guillermo de Velasco, Stephanie A. Wankowicz, Russell Madison, Siraj M. Ali, Craig Norton, Audrey Duquette, Jeffrey S. Ross, Dominick Bossé, Aly-Khan A. Lalani, Vincent A. Miller, Philip J. Stephens, Lauren Young, A. Ari Hakimi, Sabina Signoretti, Sumanta K. Pal, Toni K. Choueiri

**Affiliations:** 1 Department of Medical Oncology, Dana-Farber Cancer Institute, Boston, MA USA; 2 Department of Medical Oncology, University Hospital 12 de Octubre, Madrid, Spain; 3 Foundation Medicine, Cambridge, MA USA; 4 Memorial Sloan Kettering Cancer Center, New York, NY USA; 5 Brigham and Women’s Hospital, Boston, MA USA; 6 City of Hope, Duarte, CA USA

**Keywords:** Cancer, Renal cancer

## Abstract

**Background:**

The genomic landscape of primary clear cell renal cell carcinoma (ccRCC) has been well described. However, little is known about cohort genomic alterations (GA) landscape in ccRCC metastases, or how it compares to primary tumours in aggregate. The genomic landscape of metastases may have biological, clinical, and therapeutic implications.

**Methods:**

We collected targeted next-generation sequencing mutation calls from two independent cohorts and described the metastases GA landscape and descriptively compared it to the GA landscape in primary tumours.

**Results:**

The cohort 1 (*n* = 578) consisted of 349 primary tumours and 229 metastases. Overall, the most common mutations in the metastases were *VHL* (66.8%), *PBRM1* (41.87%), and *SETD2* (24.7%). *TP53* was more frequently mutated in metastases compared to primary tumours (14.85% versus 8.9%; *p* = 0.031). No other gene had significant difference in the cohort frequency of mutations between the metastases and primary tumours. Mutation burden was not significantly different between the metastases and primary tumours or between metastatic sites. The second cohort (*n* = 257) consisted of 177 primary tumours and 80 metastases. No differences in frequency of mutations or mutational burden were observed between primaries and metastases.

**Conclusions:**

These data support the theory that ccRCC primary tumours and metastases encompass a uniform distribution of common genomic alterations tested by next-generation sequencing targeted panels. This study does not address variability between matched primary tumours and metastases or the change in genomic alterations over time and after sequential systemic therapies.

## Introduction

Over 62,000 new cases of kidney cancers are diagnosed each year in the United States,^1^ with ~20–30% developing metastatic disease. The vast majority of metastatic cases are clear-cell renal cell carcinoma (ccRCC). Despite the availability of multiple systemic therapies, currently no tissue or blood biomarkers are used to guide systemic agents use.^2^

Next-generation sequencing (NGS) of DNA derived from tumour biopsies has greatly expanded our biological knowledge of somatic mutations in ccRCC, revealing deep genetic heterogeneity within tumours beyond VHL alteration, with several comprehensive analysis including the one conducted by The Cancer Genome Atlas (TCGA)^3,4^

In ccRCC, significantly mutated genes, beyond VHL, included tumour suppressors genes such as PBRM1, SEDT2, BAP1, or KDM5C, which remodel chromatin via histone modification(3). Few RCC mutations have potentially actionable mutations. The genomic classification of ccRCC may improve clinical management. However, the value of genomic alterations will be determined by understanding the interactions between acquired genetic alterations, treatments received, heterogeneity, and the dynamics of mutations during evolution of disease. Therefore, it is fundamental to better characterisation profile between primary and metastases not only as a means to understand variety of alterations but also as a method to develop and personalised treatments.

Studies of matched and unmatched primary and metastases have shown differences in histological grade, immunohistochemistry markers and gene expression profile, though most data have been generated based on small retrospective cohorts.^5,6^ A few small studies of paired primary tumours and metastases have also demonstrated differences in genomic alterations.^7,8^ However, the overall mutation landscape of ccRCC metastases has not been fully documented. Herein, to gain biological insights from this relationship, we describe the largest study in ccRCC comparing genomic alterations in metastases to primary tumours from non-matched tumour samples.

## Methods

### Cohort 1

Targeted NGS data were as obtained on 349 ccRCC primary tumours and 229 unmatched metastases from Foundation Medicine Inc (Cambridge, MA). ccRCC was determined based on histology, and primary tumours or metastases determined by the requisition biopsy site. Only de-identified data were used.

Targeted NGS was performed on 3769 exons from 236 cancer-related genes and 47 introns of 19 (Supplementary Table 2) commonly rearranged genes using hybridisation-captured, adaptor ligation-based libraries (Foundation Medicine, Inc.; Cambridge, MA), as previously described.^9^ SNVs were detected using a Bayesian methodology, as described in ref.^9^ Mutation calls were thrown out if the mapping quality was <25, the base calls quality score was <2, if there was evidence of strand bias (*p* < 0.001, Fisher exact test), or if the mutation was present in two or more normal controls. In addition, mutations were required to have a minor allele frequency (MAF) of at least 5% (MAF ≥ 1% at hotspots). To detect indels, the de Bruijn approach was used^9,10^ and filtered as described above. Amplifications/deletions were detected by comparing chromosomal copy number to reference normal samples. tumour mutational burden (TMB) was determined by the number of somatic mutations per megabase of targeted territory.

### Cohort 2

A validation cohort of 177 ccRCC primary tumours and 80 metastases with targeted NGS was obtained from Dana-Farber Cancer Institute (Boston, MA). All patients had ccRCC and were consented to protocol DF/HCC 11–104. Follow-up time for primary samples was determined from the time of nephrectomy to the last known date alive or death.

Targeted NGS was performed on 275 genes and intronic regions in 30 genes, for a total of 282 unique genes (Supplementary Table 2).^11^ Samples needed to have >20% tumour purity, as determined by a pathologist. Samples were aligned using the PICARD pipeline (http://broadinstitute.github.io/picard/command-line-overview.html) to GRCh37p13. Mutect^12^ and Indelocator (http://www.broadinstitute.org/cancer/cga/indelocator) were used to call SNVs and indels, respectively.

Additionally, SNVs were removed if they were present at >0.1% in Exome Variant Server^13^ [NHLBI GO Exome Sequencing Project (https://esp.gs.washington.edu/drupal/)] or present in dbSNP and appeared less than two times in the Catalogue of Somatic Mutations in Cancer (COSMIC). tumour copy number was compared to a panel of normal samples using log2 ratios. TMB was calculated as the number of somatic mutations divided by the total megabases sequenced.^14^

### Statistical analysis

Genomic alterations in primary tumours and metastases were presented descriptively. Differences in alterations were analysed using Fisher’s exact test, and corrected using Benjamini–Hochberg procedure. TMB differences were determined using Wilcoxon’s rank sum test. Statistical significance was assumed at *p* < 0.05. All statistical analysis was performed using R version 3.3.2.

## Results

### Cohort 1

**Fig. 1:**
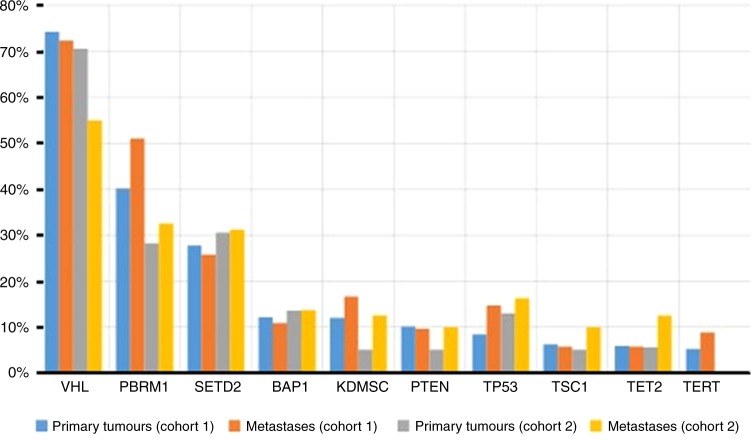
Most common genomic alterations in RCC in cohorts 1 and 2. Bar graph representing the frequency of alterations seen among primary tumours and metastases for cohorts 1 and 2

Cohort 1 consisted of 578 ccRCC patients, with 229 (41.5%) metastases and 349 (59.5%) primary tumours. Samples were from 169 women and 417 men, with a median (range) age of 58 (11–85) years (Table 1). The mean coverage was 679.3×. The most common mutations across the cohort are presented in Table 2 and Fig. 1.

**Table 1:** Clinical characteristics of cohort 1 and 2

	Cohort 1	Cohort 2
Sex		
Males	417	169
Female	169	88
Age		
Median (range)	58 (11–85)	64 (38–89)
Metastatic biopsy sites		
Lymph node	17	18
Lung	46	18
Bone	24	7
Liver	21	4
Brain	11	8
Adrenal gland	14	4
Other^a^	96	21

^a^Other includes: soft tissue, pleura

**Table 2:** Most common mutations in ccRCC in Cohorts One and Two

Genes	Cohort One							
	All Samples (*N* = 578)		Primary tumours (*N* = 349)		Metastases (*N* = 229)		*p*-value	*q*-value (BH)
	*N*	%	*n*	%	*n*	%		
*VHL*	386	66.78	261	74.79	170	74.24	0.92	1
*PBRM1*	242	41.87	136	39.19	106	47.11	0.09	0.41
*SETD2*	145	24.7	88	25.36	57	25.33	1	1
*BAP1*	80	13.8	50	14.33	30	13.1	0.71	1
*KDM5C*	75	12.97	40	11.53	35	15.56	0.21	0.63
*PTEN*	79	13.67	44	12.61	35	15.28	0.39	0.88
*TP53*	65	6.06	31	8.88	34	14.85	0.03	0.21
*TSC1*	39	6.75	23	6.59	16	6.99	0.87	1
*TET2*	29	5.02	18	5.16	11	4.8	1	1

Genes	Cohort Two							
	All Samples (*N* = 257)		Primary tumours (*N* = 177)		Metastases (*N* = 80)		*p*-value	*q*-value (BH)
	*N*	%	*n*	%	*n*	%		
*VHL*	187		125	70.62	62	77.5	0.23	0.41
*PBRM1*	76	29.57	50	28.25	26	32.5	0.56	0.72
*SETD2*	79	30.74	54	30.51	25	31.25	1	1
*BAP1*	35	13.62	24	13.56	11	13.75	1	1
*KDM5C*	19	7.39	9	5.08	10	12.5	0.15	0.41
*PTEN*	20	7.78	12	6.8	8	10	0.18	0.41
*TP53*	36	14.01	23	12.99	13	16.25	0.56	0.72
*TSC1*	17	6.61	9	5.08	8	10	0.18	0.41
*TET2*	20	7.78	10	5.65	10	12.5	0.08	0.41

Number and frequency of mutations observed across primary tumours and metastases in cohort one and two. P-values are calculated using fisher exact test and corrected using Benjamini-Hochberg.

Overall, the frequency of mutations in primary tumours and metastases were comparable (Table 2). Mutations in *TP53* were more frequent in the metastases than in the primary tumours (14.85% versus 8.9%; *p* = 0.031), however this did not pass the false discovery rate (FDR; *q* = 0.21). *PBRM1* (39.2% versus 47.1%) and *KDM5C* (11.5% versus 15.6%) were numerically more frequently mutated in metastases, but differences were not statistically significant. The median TMB for the entire cohort was 2.7 (range: 1.8–4.5). No difference in median TMB was seen between primary tumours and metastases (2.7 versus 2.7) and no difference in mutations or TMB was observed across different metastatic organ sites (Supplemental Tables 1 and  2).

### Cohort 2

Cohort 2 consisted of different 257 ccRCC patients, 177 (68.9%) primary tumours and 80 (31.1%) metastases, also from unmatched samples. Samples were from 88 women and 169 men with a median (range) age of 64 (38–89) years (Table 1). The mean target coverage was 257.64×. The most common mutations across the cohort are presented in Table 2 and Fig. 1.

No gene had a significantly different mutation frequency between primary tumours and metastases (Table 2). Similarly, there was no difference in median TMB between primary tumours and metastases (3.8 versus 4.2) (Supplemental Table 1). In cohort 2, we also performed a subset analysis comparing the frequency of gene mutations in primary tumours in patients who developed metastatic disease (*n* = 87) versus patients only had localised disease (*n* = 90) and did not develop metastases. The median follow-up time for this cohort was 21.9 months. We observed no differences in mutational frequency or TMB were observed (Table 3).

**Table 3:** Most common genomic alterations in RCC in primary tumours by metastatic or localised disease in cohort 2

	Primary tumours, localised disease (*n* = 90)		Primary tumours, metastatic disease (*n* = 87)			
Genes	*n*	%	*n*	%	*p*-value	*q*-value (BH)
	63	70	69	71.9	0.17	0.31
*VHL*	27	30	23	24	0.62	0.8
*PBRM1*	24	26.7	33	34.4	0.11	0.27
*SETD2*	7	7.8	17	17.8	0.028	0.25
*BAP1*	4	4.4	5	5.3	0.74	0.83
*KDM5C*	5	5.6	4	4.2	1	1
*PTEN*	8	8.9	15	15.6	0.12	0.27
*TP53*	2	2.2	7	7.3	0.1	0.27
*TSC1*	7	7.8	3	3.1	0.33	0.5

Number and frequency of mutations observed in primary tumours in cohort 2 between those who developed metastatic disease and those who had only localised disease. *p*-values are calculated using fisher exact test and corrected using Benjamini–Hochberg

## Discussion

To continue to make progress in identifying therapeutic targets and prognostic factors in ccRCC, it is imperative to understand the genomic landscape and differences between primary and metastatic samples. Cinically, tumour metastasis is the lethal part of cancer. Primary kidney tumour consists of cancer cells that originate from specific cell lineages. As metastatic cells originate from the primary site cell lineage, they may require specific mutations that allow them to spread and grow in distant anatomical locations, indicating that certain genomic alterations maybe enriched in the metastatic samples. If specific alterations are only observed in metastatic tumours, it would allow us to target those alterations more effectively. It is currently unknown if cohort-wide genomic alterations in RCC metastases have a different genomic profile, including potential actionable mutations, compared to samples derive from the primary site. To our knowledge, our analysis is the largest genomic ccRCC study that compares cohort-wide mutational differences between metastases and primary tumours.

Using two large ccRCC cohorts of unmatched metastases and primary tumours, we demonstrated that the genomic landscape of ccRCC metastases is similar to the genomic landscape of ccRCC primary tumours. In addition, we also established no significant genomic differences between organ sites of metastases, nor in the TMB between sites of metastases. Furthermore, in cohort 2, we revealed that there was no difference in mutations frequency in primary tumours between patients who had localised disease versus those who developed metastatic disease.

The fact that no gene(s) is overrepresented in patients who developed metastatic disease indicates that there is not a single gene driving metastatic disease in RCC. Multiple other studies have suggested non-mutation related reasons as to why a ccRCC tumour metastasises, including expression, protein, and epigenetic changes.^15–17^ However, there is emerging evidence that specific mutations in advanced RCC may increase over subsequent lines of therapy.^14^ One study of circulating free DNA from RCC patients showed increasing TP53 and mTOR pathway elements (e.g., *NF1, PIK3CA*) alterations after first-line vascular endothelial growth factor-directed therapy. This may indicate an underlying mechanism of resistance to targeted therapy, however our study was not designed to help answer this question. In addition, this study highlights that there is not a cohort-wide increase in mutations of certain genes in mRCC.

Although this is one of the largest RCC studies, there are limitations to our findings. Most important, our metastases samples were not matched with primary tumours. Therefore, we cannot draw conclusions on how an individual tumour evolves over time, especially in the presence of systemic therapies that may potentially modify the genomic profile.^18^ Future studies should utilise clonality analyses to determine alteration that may change from subclonal to clonal during metastatic progression. In addition, we were not able to describe genetic intratumoural heterogeneity, which has been shown to be a key feature in kidney cancer,^7^ and has been linked to treatment failure and drug resistance^10^ in other tumour types. Furthermore, the frequencies of mutations in most genes in both cohorts are higher than what was reported in the ccRCC cohort from TCGA,^3^ potentially due to germ line SNPs or higher clinical stage in our study. Although sequencing and mutation calling from both cohorts use well-known methods, they are not the same method and thus are not directly comparable. Another limitation of this study is that this is a retrospective study, sample enrollment was not strictly controlled, and some clinical information was not included.

In conclusion, we provide and analyse the largest metastatic ccRCC-targeting sequencing cohort to date. We demonstrate that there are no significant differences in frequencies of most common gene mutations between primary tumours and metastases from ccRCC using two different targeted gene panels.

### Data avaliability

Data from cohort 1 can be found on the Genomics Data Commons under the Foundation Medicine cohort.

## Electronic supplementary material

Supplementary Table 1(DOCX 45 kb)

Supplementary Table 2(DOCX 86 kb)
