# Supplementary material for: Targeted genomic landscape of metastases compared to primary tumours in clear cell metastatic renal cell carcinoma
Source: Br J Cancer. 2018 Apr 20;118(9):1238–42. doi: 10.1038/s41416-018-0064-3 (PMC5943584; doi:10.1038/s41416-018-0064-3)
Supplement: Supplementary file 1 — Supplementary Table 1(DOCX 45 kb) [file 41416_2018_64_MOESM1_ESM.docx]

**Supplementary Table 1. Tumor mutational burden in RCC in Cohorts One and Two**

|  | **Cohort One** | | **Cohort Two** | |
| --- | --- | --- | --- | --- |
|  | **Primary tumors** | **Metastases** | **Primary tumors** | **Metastases** |
| **Mean TMB** | 2.99 | 3.60 | 3.7 | 4.0 |
| **Median TMB (Range)** | 2.7 (0-13.5) | 2.7 (0-30.6) | 3.8 (0-11.6) | 4.2 (0-14.1) |

Supplementary Table 1: Mutational burden in metastatic and primary RCC in cohort one and two. Mean, median, and range are shown.
