# Supplementary material for: Targeted genomic landscape of metastases compared to primary tumours in clear cell metastatic renal cell carcinoma
Source: Br J Cancer. 2018 Apr 20;118(9):1238–42. doi: 10.1038/s41416-018-0064-3 (PMC5943584; doi:10.1038/s41416-018-0064-3)
Supplement: Supplementary file 2 — Supplementary Table 2(DOCX 86 kb) [file 41416_2018_64_MOESM2_ESM.docx]

**Supplementary Table 2. Mutational Breakdown by Tumor Site in Cohort One**

|  | Lung (N=46) | | Soft Tissue (N=28) | | Bone/Spine (N=24) | | Lymph Node (N=17) | | Adrenal Gland (N=14) | | Brain (N=11) | | Pleura (N=9) | |
| --- | --- | --- | --- | --- | --- | --- | --- | --- | --- | --- | --- | --- | --- | --- |
| Genes | n | **%** | **n** | **%** | **n** | **%** | **n** | **%** | **n** | **%** | **n** | **%** | **n** | **%** |
| VHL | 32 | 69.57 | 20 | 71.43 | 18 | 75 | 12 | 70.59 | 8 | 57.14 | 9 | 81.82 | 6 | 66.67 |
| PBRM1 | 21 | 47.73 | 14 | 50 | 13 | 54.17 | 7 | 41.18 | 6 | 42.86 | 7 | 63.64 | 5 | 55.56 |
| SETD2 | 10 | 22.73 | 7 | 25 | 7 | 29.17 | 4 | 23.53 | 2 | 14.29 | 1 | 9.09 | 4 | 44.44 |
| BAP1 | 5 | 10.87 | 3 | 10.71 | 9 | 37.5 | 3 | 17.65 | 1 | 7.14 | 0 | 0 | 0 | 0 |
| KDM5C | 11 | 25 | 4 | 14.29 | 1 | 4.17 | 3 | 17.65 | 3 | 21.43 | 2 | 18.18 | 2 | 22.22 |
| PTEN | 9 | 19.57 | 3 | 10.71 | 1 | 4.17 | 5 | 29.41 | 2 | 14.29 | 1 | 9.09 | 1 | 11.11 |
| TP53 | 6 | 13.04 | 5 | 17.86 | 5 | 20.83 | 4 | 23.53 | 3 | 21.43 | 1 | 9.09 | 0 | 0 |
| TSC1 | 3 | 6.52 | 2 | 7.14 | 1 | 4.17 | 0 | 0 | 0 | 0 | 1 | 9.09 | 1 | 11.11 |
| TET2 | 3 | 6.52 | 1 | 3.57 | 0 | 0 | 0 | 0 | 1 | 7.14 | 1 | 9.09 | 1 | 11.11 |
